# Changes in the nutrient content of american diets

**DOI:** 10.1186/2191-1991-1-19

**Published:** 2011-12-06

**Authors:** Kuo S Huang, Sophia Wu Huang

**Affiliations:** 1 U. S. Department of Agriculture, Economic Research Service, 355 E Street, SW, Washington, DC, 20024-3221, USA

**Keywords:** Food demand system, nutrient availabilities, nutrient demand elasticities

## Abstract

As obesity and being overweight continue to increase in the United States, public concern is growing about the quality of American diets. We compare the changes in nutrients contributed by major food groups in the periods 1953-1980 and 1981-2008 and find that there is reduced cholesterol intake and increased calcium intake, but the levels of food energy and total fats increase substantially. To understand how economic factors affect the overall nutritional quality of American diets, we estimate a complete food demand system and conduct a nutrient demand analysis. Among our findings, we conclude that some price manipulations such as subsidizing fruits and vegetables could be effective to increase produce consumption, but the effects of taxing fats to reduce the consumption of fats could be limited. Increasing income would improve intakes of nutrients such as calcium and various vitamins (likely now insufficient), but intakes of nutrients such as energy, saturated fats, and cholesterol (likely now excessive) would also rise with increased income.

## Background

The problem of obesity and being overweight in the United States has imposed heavy physical and economic toll on the Nation. Overweight and obesity are major risk factors for a number of chronic diseases such as cardiovascular disease, type 2 diabetes, hypertension, osteoporosis, and certain cancers. The U.S. Surgeon General's 2010 report indicated that about two-thirds of adults and nearly one in three children in the United States are overweight or obese, which contribute to an estimated 112, 000 preventable deaths each year [1].

The dietary pattern is a critical contributor to the recent public concern about obesity and other health problems. A poor diet and a sedentary lifestyle resulting in excessive food energy intakes could be the most important factors contributing to the problem of obesity and overweight. Also, medical evidence increasingly links excessive saturated fat and cholesterol in typical American diets with heart disease, the leading cause of death in the United States.

The issue of diet and health has become a major concern not just for consumers but also for health professionals and policy decisionmakers. The U.S. Government has advocated healthy diets through various food programs and nutrition education efforts. A notable example has been the *Dietary Guidelines for Americans *released by Dietary Guidelines Advisory Committee since 1980 [2]. These guidelines provide information and advice to help Americans make healthy food choices.

Meeting the dietary guidelines and preventing the enormous health and economic costs of obesity and overweight have motivated many researchers and concerned individuals, including public health officials, nutritionists, and economists, to investigate the causes of the obesity epidemic. For example, Gawn, etc. used income and socio-demographic variables from household survey data to explain the demand for various nutrients [3]. Drewnowski, etc. argued that relatively lower prices for refined grains, added sugar, and added fats have resulted in overconsumption of these dietary energy foods [4]. Allais, etc. assessed the effects of fat tax on the nutrients purchased by French households across different income groups and found that the nutrient effects are small and ambiguous [5]. Chouinard, etc. studied the effects of fat tax on dairy consumption and find that even a 10-percent ad valorem tax on fats would reduce the fat consumption by less than a percentage point [6].

In this study, the objective is to analyze the nutritional quality of American diets and how economic factors influence this nutrient content. At the beginning, we illustrate our answer to the question "Are Americans choosing healthier diets?" We use the available data on food consumption and the nutrient values of each food to obtain a profile of American diets and compare the changes in nutrients contributed by major food groups between 1953-1980 and 1981-2008. We then estimate a complete food demand system consisting of 13 food groups and a nonfood sector to show how food prices and income affect food consumption through the interdependent demand relationships. Finally, since changes in food consumption are likely translated into changes in the quantities of nutrients available, we incorporate the estimates of the food demand system with the information of nutrient availabilities to analyze how economic factors affect the overall nutritional quality of American diets.

## Methods

To understand the nutrient content of American diets, we focus on the structural changes in American nutritional profiles over years and showing how food prices and income affect the overall nutritional quality of American diets. We estimate a complete food demand system as a framework for nutrient analysis. The unique feature of this approach is that it incorporates all estimated price and income elasticities into the measurement of nutrient demand elasticities. Accordingly, the changes in the availability of all nutrients vary depending on how food price and income changes manifest themselves through the interdependent food demand relationships. The derivation of measurements implemented in this study is discussed below.

### Measure food nutrient availabilities

Since the unit nutrient values of each food are rather fixed because of stable food production technology, changes in the nutrient quantity are closely related to per capita food consumption, which is affected by changes in food prices or income. Consequently, let *q*_i _be the quantity of the *i*th item in a demand system of (*n-*1) foods and a nonfood sector, and *a*_ki _be the quantity of the *k*th nutrient in a total of *l *nutrients obtained from a unit of the *i*th food. The availability of a particular nutrient, say φ_k_, was calculated by multiplying per capita food consumption data across all (*n-*1) foods with the associated unit nutrient values:

(1) $${\text{φ}}_{\text{k}}={\text{Σ}}_{\text{i}}a_{\text{ki}}q_{\text{i}}\;i=\text{1},\text{2},\text{.}\;..,\left(n-\text{1}\right),k=\text{1},\text{2},...,l$$

This is what Lancaster called the "consumption technology" of consumer behavior [7]. We use this equation to transform all food consumption into nutrient availabilities and evaluate the quality of American diets over years.

### Measure food demand elasticities

It is well known that the change of a food price or consumer income will affect all foods consumed and cause a wide variety of nutrients to change simultaneously. Thus, it is desirable to estimate a complete food demand system as a framework for nutrient demand analysis. From the conceptual demand model derived from utility maximizing behavior on the part of consumers, the quantities demanded (*q*_i _'s) for (*n-*1) foods and a nonfood sector can be expressed as a function of prices (*p*_i _'s) and per capita income (*m*):

(2) $$q_{\text{i}}=f_{\text{i}}\left(p_{\text{1}},p_{\text{2}},\;...,p_{\text{n}},m\right)\;\;i=\text{ 1},\text{ 2},\;...,n$$

A first-order differential approximation to this demand equation becomes

(3) $$dq_{\text{i}}={\text{Σ}}_{\text{j}}\left(\partial q_{\text{i}}∕\partial p_{\text{j}}\right)dp_{\text{j}}+\left(\partial q_{\text{i}}∕\partial m\right)dm\;i,j=\text{1},\text{2},..,n$$

By expressing the price and income slopes in terms of elasticities, we obtain the following differential-form demand system:

(4) $$dq_{\text{i}}∕q_{\text{i}}={\text{Σ}}_{\text{j}}e_{\text{ij}}\left(dp_{\text{j}}∕p_{\text{j}}\right)+{\text{η}}_{\text{i}}\left(dm∕m\right)\;i,j=\text{1},\text{2},..,n$$

where *e*_ij _= (∂*q*_i_/∂*p*_j_)(*p*_j_/*q*_i_) is a price elasticity of the *i*th commodity with respect to a price change of the *j*th commodity, and η_i _= (∂*q*_i_/∂*m*)(*m */*q*_i_) is an income elasticity showing the effect of the *i*th quantity in response to a change in per capita income. This demand model is a general approximation of conceptual demand relationships in relating to some small change from any given point on the *n*-commodity demand surface. The merit of this approximation is that it neither imposes any rigid functional form of specification on the structure of utility function nor assumes a specific form of the demand system, for example, a double-log demand model.

This differential-form demand model is useful for empirical application. First, the demand parameters can be directly interpreted as widely used price elasticities. Other demand models, such as the Rotterdam demand system [8,9], the Almost Ideal Demand System [10], and the Translog model [11], are also capable of generating elasticities. However, their generated demand elasticities may be unstable inasmuch as they are functions of expenditure shares, which are innate stochastic variables in these models. Second, the variables in equation (4) are defined as the relative change of quantities and prices, easily quantified by using available data usually expressed in index numbers. The other demand models require the time series data of expenditure shares and are not easily available. Third, the differential-form demand model is linear in parameters for easy estimation, and this demand model is particularly useful in measuring nutrient demand elasticities as shown in the following section.

In view of classical demand theory, this differential-form demand model can be estimated by incorporating the following parametric constraints of homogeneity (Σ_j _*e*_ij _= -η_i_), symmetry (*e*_ji_/*w*_i _+ η_j _= *e*_ij_/*w*_j _+ η_i_), and Engel aggregation (Σ_i _*w*_i _η_i _= 1), where *w*_i _= *p*_i _*q*_i_/*m *is the expenditure share of *i*th commodity taken at the sample mean. The negativity condition (*e*_ii _+ *w*_i _η_i _< 0), however, is not incorporated, partly because there is no reduction in the number of parameters to be estimated and, thus, no gain in asymptotic efficiency of the estimates, and partly to avoid introducing parametric inequality constraints that would increase the complexity of estimation.

### Measure food nutrient demand elasticities

To measure the effects of changes in food prices and consumer income on nutrient availability, following Huang [12], we incorporate the demand equation (4) into the nutrient availability equation (1) as the following:

(5) $$d{\text{φ}}_{\text{k}}={\text{Σ}}_{\text{i}}a_{\text{ki}}\left[{\text{Σ}}_{\text{j}}\left(\partial q_{\text{i}}∕\partial p_{\text{j}}\right)dp_{\text{j}}+\left(\partial q_{\text{i}}∕\partial m\right)dm\right].$$

Furthermore, the relative change in nutrient availability can be expressed as a function of the relative changes in food prices and per capita income as the following:

(6) $$\begin{array}{ccc} d{\text{φ}}_{\text{k}}∕{\text{φ}}_{\text{k}} & ={\text{Σ}}_{\text{j}}\left({\text{Σ}}_{\text{i}}e_{\text{ij}}a_{\text{ki}}q_{\text{i}}∕{\text{φ}}_{\text{k}}\right)\left(dp_{\text{j}}∕p_{\text{j}}\right)+\left({\text{Σ}}_{\text{i}}{\text{η}}_{\text{i}}a_{\text{ki}}q_{\text{i}}∕{\text{φ}}_{\text{k}}\right)\left(dm∕m\right) & \\ & ={\text{Σ}}_{\text{j}}\pi_{\text{kj}}\left(dp_{\text{j}}∕p_{\text{j}}\right)+\rho_{\text{k}}\left(dm∕m\right), \end{array}$$

where π_kj _= Σ _i _*e*_ij _*a*_ki _*q*_i_/φ_k _is the nutrient-price elasticity showing the effect of a change in the *j*th food price on the availability of the *k*th nutrient, and ρ_k _= Σ _i _η_i _*a*_ki _*q*_i_/φ_k _is the nutrient-income elasticity showing the effect of a change in income on the availability of that nutrient. The estimate π_kj _represents the weighted average of all own- and cross-price elasticities (*e*_ij _'s) in response to a change in the *j*th price, with each weight expressed as the contributed share of each food to the *k*th nutrient (*a*_ki _*q*_i_/φ_k_'s). Similarly, the estimate ρ_k _represents the weighted average of all income elasticities (η_i _'s), with each weight again expressed as the contributed share of each food to the *k*th nutrient. We use the empirical estimation results based on equation (6) to analyze how food prices and income affecting nutrient availabilities.

### Changes in Nutrient Availabilities

For several decades, the efforts of Federal nutrition education in the United States have focused on providing consumers with information to help Americans make healthy food choices. *The 2010 Dietary Guidelines for Americans *encourage increased consumption of high-fiber whole-grain products, fat-free or low-fat milk, and a variety and sufficient amount of fruits and vegetables. The consumption of fats and oils as part of a healthful diet should come from sources of poly- and mono-unsaturated fatty acids such as fish, nuts, and vegetable oils, while selecting and preparing meat and poultry should be lean to avoid excessive intakes of high-saturated fatty acids. Also, the guideline recommends that foods and beverages should be selected and prepared with little added sugar or caloric sweeteners. For a better understanding as to whether Americans are following these dietary guidelines to choose healthier diets, we analyze the changes in daily nutrient levels consumed by an average American over years.

#### Data

The per capita food consumption data are compiled from the Economic Research Service's *Food Consumption Data System *[13] with a total of 131 food items. The nutrient values of each food item for these 131 foods are compiled from the Agricultural Research Service's *National Nutrient Database for Standard Reference *[14]. We multiply the quantity of each food item with its corresponding nutrient values to derive the nutrient availabilities in American diets for all 131 food items from 1953 to 2008. In this study, we focus on 12 major nutrients, encompassing three nutrient categories, namely macronutrients (energy, protein, total fats, saturated fat, cholesterol, and dietary fiber), minerals (calcium and iron), and vitamins (vitamin C, folate, vitamin A, and vitamin E).

To make this huge data set manageable for presentation, we aggregate the per capita nutrient availabilities of the 131 food items into 13 food groups by summing up nutrient values of each individual food. These 13 food groups are (1) the meat group, including beef, veal, and pork; (2) the poultry group, including chicken and turkey; (3) the fish group, including fresh, frozen, and canned fish; (4) the egg group; (5) the dairy group, including milk and dairy products; (6) the fat group, including added fats of butter, margarine, and other fats and oils; (7) the fresh fruit group; (8) the fresh vegetable group; (9) the processed produce group, which also includes fruit and vegetable juices and tree nuts; (10) the wheat flour group; (11) the starch group, including potato, rice, corn flour and oat products; (12) the sugar group, including all added sugars and other sweeteners; and (13) the nonalcoholic beverage group, including coffee, tea and cocoa, but not including other drinks like carbonated beverages, sports drinks, fruit drinks, and other sweetened fruit flavored drinks for lack of consistent times series for these products.

**Figure 1:**
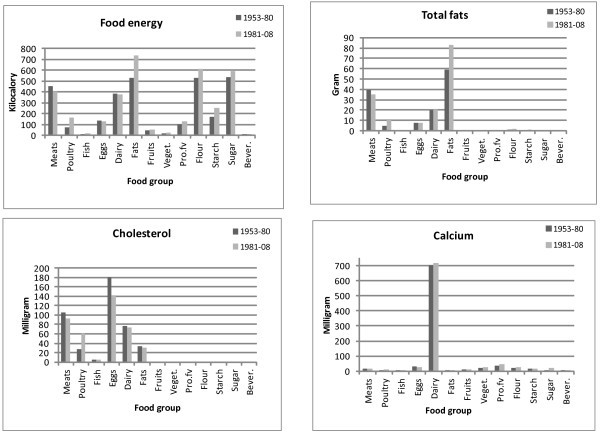
**Selected daily per capita nutrients between periods 1953-1980 and 1981-2008**.

Thus, from 1953 to 2008, we have a matrix of 12 by 13 nutrient availabilities for each year to portray the daily nutrient diets of an average American. By comparing the nutrient availabilities of 1953-1980 against those of 1981-2008, we calculate the average nutrient values for each period as shown in table 1. In addition, to show the changes of nutrients on those of currently public health concerns on food energy, total fat, cholesterol, and calcium, we depict their nutrient availabilities between the two periods in Figure 1. The highlights of our major findings follow:

**Table 1:** Changes in daily per capita nutrient values between 1953-1980 (A) and 1981-2008 (B)

Nutrient	Period	**Nutrient **		Meats	Poultry	Fish	Eggs	Dairy	Fats	Fruits	Veget.	Pro.fv	Flour	Starch	Sugar	Bever.
		**value**	**unit**				**Changes in nutrient values**									

Energy	A	2989.5	Kcal	451.3	74.6	12.7	62.8	384.2	526.8	44.4	18.9	173.0	530.6	168.2	533.0	9.0
	B	3504.4		406.7	165.8	15.5	49.5	379.8	735.4	53.0	24.9	204.6	610.8	253.2	593.1	12.1

Protein	A	83.2	G	22.8	7.0	2.1	5.4	20.6	0.1	0.5	0.8	4.1	15.0	4.0	0.0	0.7
	B	94.7		20.2	15.4	2.6	4.2	21.8	0.1	0.6	1.1	5.1	17.3	5.2	0.0	1.0

Total fat	A	135.2	G	39.2	5.0	0.4	4.2	20.1	59.1	0.3	0.1	4.0	1.4	0.7	0.0	0.6
	B	162.6		35.5	11.1	0.5	3.3	20.6	82.6	0.5	0.2	4.9	1.7	1.0	0.0	0.8

Saturated fat	A	50.7	G	15.0	1.4	0.1	1.3	12.5	19.1	0.1	0.0	0.5	0.2	0.1	0.0	0.3
	B	56.0		13.5	3.2	0.1	1.0	12.9	23.6	0.1	0.0	0.6	0.3	0.2	0.0	0.4

Cholesterol	A	429.7	Mg	105.1	27.7	4.5	180.8	77.2	34.3	0.0	0.0	0.0	0.0	0.0	0.0	0.0
	B	405.7		93.3	61.5	5.5	142.5	72.8	30.2	0.0	0.0	0.0	0.0	0.0	0.0	0.0

Dietary fiber	A	14.2	G	0.0	0.0	0.0	0.0	0.1	0.0	1.8	1.3	3.0	4.1	2.8	0.0	1.2
	B	16.1		0.0	0.0	0.0	0.0	0.1	0.0	2.0	1.6	3.5	4.6	2.7	0.0	1.7

Calcium	A	880.3	Mg	17.5	4.3	4.8	22.7	703.1	4.0	11.1	19.4	44.7	22.0	15.7	6.0	4.9
	B	927.2		15.6	9.6	3.9	17.8	718.6	3.2	10.7	24.1	54.7	25.2	16.2	20.9	6.7

Iron	A	14.2	Mg	1.9	0.4	0.1	0.8	0.4	0.0	0.2	0.3	1.6	6.7	1.1	0.2	0.5
	B	16.7		1.7	0.9	0.1	0.6	0.4	0.0	0.2	0.4	2.1	7.8	1.1	0.7	0.7

Vitamin C	A	82.2	Mg	0.2	0.5	0.0	0.0	4.4	0.0	16.2	7.9	36.4	0.0	16.6	0.0	0.0
	B	87.6		0.2	1.1	0.0	0.0	3.6	0.0	16.5	11.3	43.8	0.0	11.0	0.0	0.0

Folate	A	220.7	Mcg	8.9	3.5	0.9	20.1	27.0	0.3	10.5	28.7	58.6	37.8	22.3	0.6	1.5
	B	246.4		7.5	7.5	0.9	15.8	24.9	0.2	12.0	35.8	66.7	43.6	26.8	2.6	1.9

Vitamin A	A	561.6	RE	1.6	19.6	2.3	59.8	216.8	156.5	7.8	57.7	38.0	0.0	1.5	0.0	0.0
	B	610.1		1.5	42.9	2.8	47.1	266.2	126.8	6.9	75.6	36.9	0.0	3.3	0.0	0.0

Vitamin E	A	8.4	ATE	0.3	0.1	0.1	0.4	0.4	5.1	0.2	0.2	1.3	0.1	0.1	0.0	0.0
	B	12.7		0.3	0.3	0.1	0.3	0.4	9.0	0.3	0.2	1.6	0.1	0.1	0.0	0.0

Notes: Kcal = kilocalories/calories, G = grams, Mg = miligrams, Mcg = micrograms, RE = retinol equivlents and ATE = alpha-tocopherol equivalents. Nutrient values are based on 131 selected food items. Total fat refers to saturated and unsaturated fatty acids. Fats group refers to butter, margarine, and cooking oils; Veget. is vegetables; Pro.fv is processed fruits and vegetables; Bever. is nonalcoholic beverages. Period A represents 1953-1980, and period B represents 1981-2008.

#### Macronutrients

Food energy--as shown in table 1, excess intakes of food energy are a serious public health problem in the United States. The daily food energy availability per person between the two periods increased 17 percent or 514 calories from 2, 989.5 to 3, 504.4 calories. The increase of food energy reflects American increased consumption of some energy-yielding food groups, such as the fat, poultry, flour, and starch groups. In particular, energy from the fat group, increasing 208 calories from 527 to 735 calories, contributed the most to the upsurge of food energy. Poultry products also contributed an increase of energy by 91.2 calories. The energy from the meat group, however, decreased by 44.6 calories.

Protein--the daily protein availability increased 14 percent from 83.2 to 94.7 grams between the two periods. The main food group responsible for the increase was the poultry group; its protein contribution increased from 7 to 15.4 grams. The protein from the meat group, however, showed a decrease from 22.8 to 20.2 grams.

Total fat--American daily per capita availability of total fat increased from 135.2 to 162.6 grams between the two periods. Most of the increase came from the fat group of foods, responsible for an increase of total fat by 23.5 grams from 59.1 to 82.6 grams. Other major food groups contributed to the nutrient of total fat including meats and poultry products. But the total fat from meats decreased slightly from 39.2 to 35.5 grams, while that from the poultry group increased more than double, from 5 to 11.1 grams between the two periods.

Saturated fat--similar to total fat, the daily availability of saturated fat increased 5.3 grams from 50.7 to 56 grams per person. The fat group of foods contributed the most to the increase by 4.5 grams from 19.1 to 23.6 grams, and the poultry group gave another increase of 1.8 gram.

Cholesterol-- the daily level of cholesterol in the American diet declined 6 percent or 24 milligrams from 429.7 to 405.7 milligrams per person between the two periods. Much of cholesterol comes from the food groups of eggs, meats, and dairy products; the amount of cholesterol contributed from these groups was reduced substantially. For example, reduced food consumption from the egg group caused its contribution to cholesterol availability to decrease by 38.3 milligrams from 180.8 to 142.5 milligrams. Similarly, the meat group also contributed less to the level of cholesterol by 11.8 milligrams from 105.1 to 93.3 milligrams.

Dietary fiber--the daily level of dietary fiber in the United States increased from 14.2 to 16.1 grams per person between the two periods, with wheat flour--the leading source of dietary fiber--contributing most of the increase, from 4.1 to 4.6 grams. Other food groups, including fruits, vegetables and processed produce, also slightly increased their contributions about 0.2 to 0.5 grams.

#### Minerals

Calcium--as the main nutrient in the mineral category, the per capita daily calcium levels in U.S. food consumption are quite stable between the two periods, with a slight increase from 880.3 to 927.2 milligrams. Increased consumption of the dairy group, the dominant source of calcium, contributed to a slight boost of American daily calcium availabilities from 703.1 to 718.6 milligrams between the two periods.

Iron--American daily per capita availability of iron increased 2.5 milligrams from 14.2 to 16.7 milligrams between the two periods. This increase mainly came from the food groups of wheat flour and poultry products, 1.1 milligrams and 0.5 milligrams, respectively.

#### Vitamins

Vitamin C--almost all vitamin C came from the food groups of fruits, vegetables, and processed produce with fruit juices being the main source. The level of American daily availability of vitamin C increased from 82.2 to 87.6 milligrams between the periods. This 7-percent increase was mainly due to the increased contributions from the vegetable and processed produce groups, 3.4 and 7.4 milligrams, respectively.

Folate --the daily level of folate (a B-vitamin) increased 25.7 micrograms from 220.7 to 246.4 micrograms per person between the two periods. The major contributors to this increase came mainly from the food groups of vegetables, processed produce, and flour, with a range of 5 to 7 micrograms.

Vitamin A--vitamin A can be found in large amounts from the food groups of dairy products and fats. The daily level in U.S. food consumption increased from 561.6 to 610.1 retinol equivalents (RE) per person between the periods. This 9-percent increase, however, showed significant shifts in its sources decreasing from the fat and the egg groups but increasing from the dairy and the vegetable groups.

Vitamin E--the daily availability of vitamin E in American diets between the two periods increased from 8.4 to 12.7 ATE (alpha-tocopherol equivalents) per person; the main source of increase came from the fat group, with an increase of 3.9 ATE from 5.1 to 9 ATE.

In summary, Americans appear to be trending toward more healthful diets as measured by a reduction in cholesterol intake and an increase in the availabilities of protein, dietary fiber, calcium, iron and various vitamins. But Americans still need to make considerable efforts to reduce their intake levels for food energy, total and saturated fatty acids, because excessive intakes of total and saturated fatty acids are associated with elevated blood cholesterol levels, a risk factor for coronary heart disease.

It should be noted that the nutrient availability data used in this study are measured at the aggregate level, based on foods in their commodity forms, and may not be accurate reflections of the nutrient changes that would occur at the consumer level. These food availability data are unable to take into account food preparation methods, which can heavily influence the final nutrient content of foods. For example, whether the chicken is fried or roasted and whether the skin is eaten considerably affects the final nutritional characteristics of the chicken consumed. Similarly, although grain products are naturally low in fat, preparation methods that incorporate added fats could result in high-fat content for many grain food products, such as baked goods. Also, the food availability data are slow in measuring and reflecting changes in the nutrient composition of the commodities themselves, such as for lean meat and increasing availability of lower-fat cheeses, and, therefore, may not accurately reflect the current nutrient contribution of each food group to each total nutrient.

### Food Prices and Income Affect Food Consumption

In the consumer budgeting process, a complete food demand system to reflect the interdependent demand relationships among all foods is important for nutritional analysis. For example, if the price of beef goes up while the price of chicken remains the same, consumers will likely buy less of the relatively more expensive beef and buy more of the relatively less expensive chicken. Consumption of other foods could also be affected. If consumers buy less beef, such as hamburger meat, they might also buy less cheese and fewer hamburger rolls because of their complementary uses in cheeseburgers. Because different foods provide different nutritional profiles, a change in beef price or consumer income will likely affect changes in the foods purchased, thereby translating into the quantities of nutrients available in consumer diets. Thus the estimates of a complete food demand system are essential for providing basic input information in the analysis of how food prices and income affect nutrient availabilities.

#### Data

We estimate a complete food demand system based on equation (4) for 13 food groups and a nonfood sector. The data required for the estimation are quantities, prices, income, and expenditure shares. The raw quantity data for per capita food consumption consisting of 131 food items covering 1953-2008 are compiled from the Economic Research Service's *Food Consumption Data System*. These quantity data are then aggregated into 13 food groups as defined in the previous section by using the Laspeyres indexes.

The corresponding price indexes for these food groups, which are components of the consumer price index (CPI) with a base of 1982-84 = 100, are obtained from the Bureau of Labor Statistics [15]. Per capita income is approximated by per capita personal expenditures, obtained from the Bureau of Economic Analysis [16]. The quantity index for the nonfood sector is calculated from the current value of per capita expenditure on nonfood divided by the CPI of all items less food. The average expenditure shares between food and nonfood sectors in 1982-84 are calculated from the personal consumption expenditures. Given the expenditure share of total food, this share is proportionally allocated to each individual food group in accordance with its value in 1982-84.

The empirical estimates of the food demand system consisting of 13 food groups and a nonfood sector are presented in table 2. The quantities are listed in the left column with respect to their prices, per capita income listed at the top of the table. For each pair of estimates, the upper part is the estimated elasticity, and the lower part the estimated standard error. We can easily verify that all estimated elasticities in the table satisfy the theoretical constraints of symmetry, homogeneity, and Engel aggregation. The expenditure shares are listed at the bottom of the table. To represent the goodness of fit, the common measure *R*^2^, however, is not applicable for this study because all demand equations are estimated simultaneous with parametric constraints. We therefore calculate the root-mean-square (*RMS*) percentage errors of the *ex post *simulation to sample means of actual observations to represent the goodness of fit for each demand equation. Most of estimated *RMS *errors are less than 5 percent for each demand equation.

**Table 2:** U.S. food demand system (estimated elasticities), 1953-2008

Quantity	Price of each food or nonfood group														Income
		
	Meats	Poultry	Fish	Eggs	Dairy	Fats	Fruits	Veget.	Pro.fv	Flour	Starch	Sugar	Bever.	N.food	
Meats	-0.4599	0.0608	0.0494	-0.0006	-0.0037	0.0051	-0.0652	-0.0237	0.1848	0.0072	-0.0499	0.0015	-0.0063	-0.1325	0.4330
	0.0487	0.0198	0.0255	0.0038	0.0183	0.0287	0.0329	0.0214	0.0627	0.0401	0.0274	0.0114	0.0175	0.1198	0.1163

Poultry	0.2201	-0.4631	0.0011	0.0148	0.0463	0.0897	0.0870	0.0264	-0.1209	-0.1435	0.0668	-0.0996	-0.0261	0.0044	0.2964
	0.0681	0.0675	0.0673	0.0131	0.0543	0.0647	0.0617	0.0559	0.1202	0.0947	0.0625	0.0338	0.0365	0.1906	0.1886

Fish	0.2167	-0.0041	-0.1420	-0.0435	-0.2785	-0.2650	-0.0037	-0.0164	0.0667	-0.1042	0.1103	0.1508	0.0108	-0.5048	0.8068
	0.1176	0.0899	0.1813	0.0237	0.1069	0.1160	0.1046	0.1069	0.2018	0.1723	0.1129	0.0648	0.0677	0.3131	0.3063

Eggs	0.0050	0.0621	-0.1274	-0.0930	-0.0536	0.0482	-0.0066	0.0175	0.0496	0.1134	-0.0375	-0.1108	0.0148	0.0235	0.0950
	0.0532	0.0542	0.0729	0.0277	0.0761	0.0583	0.0472	0.0599	0.0939	0.0949	0.0568	0.0424	0.0309	0.1384	0.1372

Dairy	0.0022	0.0241	-0.1001	-0.0069	-0.0167	-0.0838	0.0100	0.0091	0.0690	-0.0216	-0.0150	0.0326	0.0314	-0.1371	0.2027
	0.0316	0.0273	0.0401	0.0093	0.0508	0.0330	0.0282	0.0319	0.0558	0.0524	0.0317	0.0217	0.0181	0.0833	0.0804

Fats	0.0194	0.1387	-0.3176	0.0174	-0.2773	-0.0352	-0.2146	-0.0173	0.8612	-0.5513	-0.0797	-0.0964	-0.1196	-0.0004	0.6724
	0.1619	0.1046	0.1403	0.0229	0.1060	0.2077	0.1493	0.1190	0.2996	0.2135	0.1369	0.0686	0.0889	0.4851	0.4688

Fruits	-0.1665	0.0664	0.0025	-0.0016	0.0145	-0.0989	-0.4156	0.0453	-0.3182	0.2879	0.0015	0.0566	0.0491	0.2440	0.2329
	0.0884	0.0475	0.0602	0.0089	0.0433	0.0706	0.1106	0.0526	0.1567	0.0989	0.0667	0.0277	0.0435	0.2932	0.2790

Veget.	-0.0950	0.0223	-0.0118	0.0028	0.0077	-0.0112	0.0540	-0.2132	-0.0354	-0.3748	0.1693	0.0234	-0.0214	-0.2248	0.7081
	0.0751	0.0569	0.0814	0.0148	0.0645	0.0749	0.0695	0.0938	0.1315	0.1113	0.0791	0.0401	0.0428	0.2030	0.2039

Pro.fv	0.3125	-0.0703	0.0239	0.0038	0.0530	0.2759	-0.2284	-0.0216	-1.4250	-0.0700	-0.1904	-0.0384	0.1133	0.2085	1.0531
	0.1154	0.0624	0.0783	0.0118	0.0578	0.0960	0.1067	0.0673	0.2961	0.1312	0.0892	0.0362	0.0568	0.4760	0.4583

Flour	0.0171	-0.0493	-0.0228	0.0095	-0.0154	-0.1180	0.1335	-0.1242	-0.0301	-0.1334	0.0894	0.0872	-0.0067	-0.0523	0.2156
	0.0500	0.0338	0.0460	0.0082	0.0372	0.0471	0.0458	0.0389	0.0902	0.0900	0.0421	0.0228	0.0260	0.1439	0.1364

Starch	-0.2636	0.1386	0.1693	-0.0137	-0.0258	-0.0797	0.0236	0.3275	-0.6346	0.5127	-0.4604	0.2169	-0.1358	1.3992	-1.1742
	0.1784	0.1172	0.1584	0.0258	0.1183	0.1583	0.1626	0.1459	0.3212	0.2215	0.2095	0.0742	0.0937	0.5398	0.5306

Sugar	0.0233	-0.1541	0.1858	-0.0430	0.1065	-0.0912	0.1208	0.0436	-0.0964	0.3983	0.1787	-0.2741	-0.0206	-0.4141	0.0363
	0.0633	0.0543	0.0777	0.0165	0.0691	0.0682	0.0577	0.0632	0.1124	0.1027	0.0635	0.0573	0.0366	0.1659	0.1631

Bever.	-0.0392	-0.0432	0.0148	0.0045	0.0904	-0.1160	0.0967	-0.0312	0.3528	-0.0401	-0.1247	-0.0238	-0.4157	-0.2737	0.5484
	0.0966	0.0573	0.0796	0.0118	0.0567	0.0863	0.0892	0.0661	0.1713	0.1143	0.0787	0.0358	0.0714	0.2900	0.2792

N.food	-0.0327	-0.0089	-0.0076	-0.0027	-0.0236	-0.0031	-0.0086	-0.0074	0.0036	-0.0301	-0.0035	-0.0109	-0.0063	-0.9943	1.1362
	0.0034	0.0016	0.0022	0.0003	0.0016	0.0025	0.0032	0.0018	0.0070	0.0034	0.0024	0.0009	0.0016	0.0173	0.0156

Expend.	0.0378	0.0107	0.0081	0.0026	0.0214	0.0067	0.0142	0.0106	0.0208	0.0306	0.0058	0.0068	0.0069	0.8170	

Notes: For each pair of estimates, the upper part is the estimated elasticity, and the lower part is the standard error. Veget. is vegetables; Pro.fv is processed fruits and vegetables; Bever. is nonalcoholic beverages; N.food is nonfood; Expend. is expenditure shares.

#### Quantity responses to changes in prices

The estimated own-price elasticities are listed in the diagonal entries of table 2. All estimates are negative signs as expected, and most are statistically significant with t-ratios (the ratios of estimated coefficients to standard errors) greater than two. The own-price elasticities of meats, poultry products, fruits, starch foods, and nonalcoholic beverages are around -0.45. They indicate that, holding the same prices of all other groups and per capita income, a marginal 10-percent increase in the price of an individual food group would reduce its quantities demanded about 4.5 percent.

The elasticity of processed produce is relatively price elastic at -1.425. It is plausible that the processed produce can be stored for a long time and consumers would purchase a significant quantity during a sale period. On the contrary, the elasticity of the vegetable group is relatively price inelastic at -0.2132 because fresh vegetables are highly perishable, and consumers have less flexibility in adjusting their quantities purchased in response to price changes. The price elasticities of fish, dairy products, and fats and oils, however, are not statistically significant, probably because of difficulty in defining prices and quantities to match closely for such a wide variety of food items contained in each food group.

Public and private sector nutritionists have increasingly emphasized the need for Americans to increase their consumption of fruits and vegetables. Our estimated price elasticities for fruits (-0.4156), vegetables (-0.2132), and processed produce (-1.425) would suggest that a price reduction could be effective in increasing produce consumption. On the contrary, some policy decisionmakers are considering reducing fat intakes in American diets by imposing taxes on the fat in food. Our estimated price elasticities for fats and oils (-0.0352) and dairy products (-0.0167) are relatively price inelastic and statistically insignificant, and thus the effect of taxing fats to reduce consumption could be limited.

The estimated cross-price elasticities reflecting the interdependent demand relationships of food consumption are listed in the off-diagonal entries of the table. These elasticities reflect the consumers' view of substitute or complementary relationships of certain price changes depending on the sign being positive or negative. For example, the cross-price elasticity of meats with respect to the price change of poultry products is 0.0608, implying substitution relationships between these two food groups. A marginal 10-percent increase in the price of poultry products would reduce the quantities demanded for poultry products but would cause the quantities demanded for meats to increase by 0.6 percent because of substituting meats for poultry. On the contrary, the cross-price elasticity of meats with respect to the price change of the starchy food group (mainly potatoes) is -0.0499. A marginal increase in the price of the starchy food group would reduce the quantities demanded for both meats and the starchy foods because of their complementary relationships.

#### Quantity responses to changes in income

The estimated income elasticities are listed in the column under "income." Most of the estimated income elasticities are statistically significant and show positive signs as expected. For example, the estimates are 0.433 for meats and 0.2964 for poultry products showing that a 10-percent increase in per capita income would increase their quantities demanded by 4.3 and 3 percent, respectively. The income elasticities for the groups of fish and processed produce are relatively elastic, respectively, at 0.8068 and 1.0531. The income elasticity of the starchy group, however, shows a negative sign implying that it is an inferior food group, mainly potatoes.

### Food Prices and Income Affect Nutrient Availabilities

Given the nutrient shares of individual food groups calculated from table 1 and a complete set of all price and income elasticities obtained from table 2, we calculate the nutrient responses to changes in food prices and per capita income based on equation (6). As discussed earlier, the magnitude of nutrient responses to a price change for any particular food group is estimated as the weighted average of all own- and cross-price elasticities, with each weight expressed as the contributed share of each food to a particular nutrient. Since the current status of American diets is our primary concern, we calculate the nutrient demand elasticities of the food group in 1981-2008 by using the average nutrient share of that period. Similarly, the nutrient responses to income can be estimated as the same weighted average of all income elasticities. In addition, we have set those insignificant cross-price elasticities in the demand system as zero for the calculation of nutrient elasticities.

#### Nutrient responses to changes in prices

As shown in table 3, the upper part of the table presents the nutrient shares of 12 nutrients for all 13 food groups in 1981-2008. The lower part shows the percentage change in the availability of 12 nutrients in response to a marginal increase in the price of any one food group by 10-percent (holding the prices of other food groups constant) or to a 1-percent increase in per capita income. Taking meat group as an example, the group contributes the nutrient shares for energy at 11.61 percent, saturated fats 24.17 percent, cholesterol 22.99 percent, and iron 10.39 percent. Also, as shown in the lower part of the table, the net effects of a 10-percent increase in the price of the meat group would reduce daily per capita availability of energy by 0.46 percent or equivalent 16.12 calories on the basis of a total 3, 504 calories. Other nutrients would also be reduced: saturated fat by 0.96 percent (0.54 gram), cholesterol by 0.69 percent (2.82 milligrams), and iron by 0.18 percent (0.03 milligrams).

**Table 3:** Nutrient shares and their economic responses by food groups, 1981-2008

Nutrient			Meats	Poultry	Fish	Eggs	Dairy	Fats	Fruits	Veget.	Pro.fv	Flour	Starch	Sugar	Bever.	Total
	**Value**	**Unit**	**Nutrient share of each food group (percent)**													

Energy	3504.4	Kcal	11.61	4.73	0.44	1.41	10.84	20.98	1.51	0.71	5.84	17.43	7.22	16.92	0.34	100
Protein	94.7	G	21.28	16.26	2.78	4.47	23.01	0.11	0.66	1.12	5.41	18.29	5.52	0.00	1.07	100
Total fat	162.6	G	21.84	6.85	0.29	2.06	12.66	50.78	0.32	0.11	3.00	1.01	0.60	0.00	0.47	100
Saturated fat	56.0	G	24.17	5.66	0.21	1.86	23.01	42.22	0.17	0.06	1.15	0.47	0.30	0.00	0.74	100
Cholesterol	405.7	Mg	22.99	15.15	1.35	35.12	17.95	7.43	0.00	0.00	0.00	0.00	0.00	0.00	0.00	100
Dietary fiber	16.1	G	0.00	0.00	0.00	0.00	0.32	0.00	12.42	9.95	21.66	28.64	16.46	0.01	10.54	100
Calcium	927.2	Mg	1.68	1.03	0.42	1.93	77.51	0.35	1.15	2.59	5.90	2.72	1.75	2.25	0.72	100
Iron	16.7	Mg	10.39	5.13	0.63	3.69	2.56	0.08	1.17	2.16	12.45	46.55	6.74	4.18	4.26	100
Vitamin C	87.6	Mg	0.22	1.24	0.05	0.00	4.10	0.01	18.84	12.95	50.07	0.00	12.52	0.01	0.00	100
Folate	246.4	Mcg	3.06	3.06	0.38	6.42	10.11	0.09	4.86	14.53	27.08	17.69	10.88	1.07	0.75	100
Vitamin A	610.1	RE	0.24	7.03	0.46	7.73	43.63	20.79	1.13	12.40	6.04	0.00	0.54	0.00	0.00	100
Vitamin E	12.7	ATE	2.28	2.00	0.66	2.57	3.18	70.68	2.18	1.94	12.69	0.83	0.94	0.00	0.05	100

			**Nutrient responses of a 10-percent price increase or a 1-percent income increase (percent)**													**Income**

Energy	3504.4	Kcal	-0.46	-0.13	-0.31	-0.06	-0.43	-0.35	-0.24	-0.02	0.71	-0.38	0.00	-0.39	-0.26	0.25
Protein	94.7	G	-0.56	-0.64	-0.13	-0.01	-0.11	-0.21	0.10	-0.12	-0.74	-0.17	-0.19	0.17	0.02	0.26
Total fat	162.6	G	-0.78	0.52	-1.65	-0.01	-1.43	-0.17	-1.24	-0.05	4.32	-2.85	-0.14	-0.52	-0.56	0.51
Saturated fat	56.0	G	-0.96	0.48	-1.47	-0.01	-1.21	-0.28	-1.03	-0.05	4.01	-2.38	-0.12	-0.40	-0.45	0.47
Cholesterol	405.7	Mg	-0.69	-0.24	-0.77	-0.31	-0.27	-0.08	-0.18	-0.05	1.01	-0.23	-0.01	-0.53	-0.03	0.24
Dietary fiber	16.1	G	-0.06	0.02	0.28	0.03	0.09	0.01	-0.53	-0.03	-4.15	0.45	-0.88	0.59	-0.35	0.23
Calcium	927.2	Mg	0.05	-0.08	-0.74	-0.03	-0.12	-0.56	-0.12	-0.04	-0.38	0.07	-0.09	0.21	0.26	0.24
Iron	16.7	Mg	-0.18	-0.43	0.16	0.00	0.06	-0.30	0.35	-0.43	-1.93	-0.19	-0.09	0.32	-0.11	0.26
Vitamin C	87.6	Mg	0.82	-0.11	0.17	0.00	-0.01	1.17	-1.92	0.13	-8.51	0.68	-1.30	0.19	0.50	0.49
Folate	246.4	Mcg	0.28	-0.19	0.03	-0.04	-0.01	0.40	-0.56	-0.18	-4.58	-0.02	-0.60	0.22	0.18	0.35
Vitamin A	610.1	RE	0.19	-0.02	-1.19	-0.06	-0.66	-0.23	-0.57	-0.25	1.08	-1.56	0.12	-0.21	-0.04	0.40
Vitamin E	12.7	ATE	0.27	0.85	-2.29	-0.02	-1.98	0.04	-1.88	-0.03	4.19	-3.87	-0.24	-0.72	-0.70	0.64

Notes: Kcal = kilocalories/calories, G = grams, Mg = miligrams, Mcg = micrograms, RE = retinol equivlents and ATE = alpha-tocopherol equivalents. Nutrient values are based on 131 selected food items. Total fat refers to saturated and unsaturated fatty acids. Fats group refers to butter, margarine, and cooking oils; Veget. is vegetables; Pro.fv is processed fruits and vegetables; Bever. is nonalcoholic beverages.

Although the meat group contributes little to various vitamins, a 10-percent price increase for this group would increase the availability of vitamin C by 0.82 percent (0.71 milligram), vitamin A by 0.19 percent (1.17 RE), and vitamin E by 0.27 percent (0.03 ATE). This is because, as shown in table 2, an increase in the price of the meat group is associated with increased consumption of other food groups such as the fats and oils (rich in vitamins A and E) and processed produce (rich in vitamin C). This example highlights the importance of interdependent demand relationships among the different food groups through cross-price effects.

The following highlights illustrate nutrient responses to price increases for those nutrients that are current public health concerns--excessive intake levels for food energy, total fat, cholesterol, and intake level shortfalls for calcium:

Food energy--the availability of food energy mainly comes from the fat group by 20.98 percent in the form of total fat. The flour group contributes 17.43 percent in the form of protein and carbohydrate. The sugar group contributes 16.92 percent in the form of carbohydrate. Meats and poultry products contribute another 16.34 percent in the form of protein and total fat. A 10-percent price increase for each food group of meats, dairy, and flour would reduce daily per capita energy availability about 0.4 percent or the equivalent of 14 calories.

Saturated fats--the saturated fats come mainly from the food groups of fats by 42.22 percent, meats by 24.17 percent, and dairy by 23.01 percent. The effect of a 10-percent price increase for the fat group would reduce daily per capita saturated fat availability by only 0.28 percent, probably because the commodities included in the fat group are used mostly for added fats in food preparations and therefore not sensitive to its own price changes. The commodities in the fat group, however, are complementary with wheat flour (cross-price elasticity -0.5513 in Table 2) for preparing foods, such as bakery products. Thus, while the same price increases for flour would reduce flour consumption, the price increase would also reduce saturated fat availability by 2.38 percent.

Cholesterol-- cholesterol is found only in animal products, and the major source of cholesterol comes from the egg group by 35.12 percent, because eggs contain an exceptionally high level of cholesterol, 1, 639 milligrams per pound. The remaining cholesterol consumed comes from meats by 22.99 percent, poultry by 15.15 percent, and dairy by 17.95 percent. The effects of a 10-percent increase in the price of eggs would reduce per capita cholesterol consumption by only 0.31 percent. Since eggs include fresh and processed uses, many eggs are sold primarily to food manufacturers for processed foods such as candy and baked goods, and thus the contained cholesterol is not sensitive to retail price changes for eggs. The same price increase in meats, poultry, fish and dairy would reduce cholesterol intake in a range of 0.24 and 0.77 percent.

Calcium--it comes mostly from dairy products with a share of 77.51 percent. For all other calcium sources, each food group provides less than 5 percent. The increase in dairy price by 10-percent, however, affects little decrease in the availability of calcium by 0.12 percent, probably because consumer demand for calcium depends heavily on popular calcium supplements instead of consuming dairy products, which contain high levels of saturated fat and cholesterol. However, a 10-percent price increase in either fish or fats would reduce the availabilities of calcium by 0.74 and 0.56 percent, respectively.

#### Nutrient responses to changes in income

The net effects of changes in nutrient availability caused by an increase in per capita income are listed in the last column of the lower part of table 3. According to the estimates, an increase of consumer income by 1 percent would increase energy by 0.25 percent, protein by 0.26 percent, total fat by 0.51 percent, saturated fats by 0.47 percent, and cholesterol by 0.24 percent. The same income increase would increase calcium by 0.24 percent, iron by 0.26 percent and vitamin C by 0.49 percent. Obviously, the net nutritional effects of increasing consumer income are mixed. Increased income would increase consumption of nutrients currently consumed in low amounts, such as calcium and iron. But it would also increase the consumption of other nutrients, such as total fat, saturated fats, and cholesterol, which are already consumed in excessive amounts.

## Conclusions

As the rates of obesity and being overweight continue to increase in the United States, public concern is growing about the quality of American diets. By comparing the nutrient availabilities between 1953-1980 and 1981-2008, we find that American nutritional status appears to be trending toward healthier diets as measured by a reduction in cholesterol intakes and an increase in the intakes of protein, dietary fiber, calcium, iron, and various vitamins. The levels of food energy, total fats and saturated fats, however, also increased substantially and likely caused the prevalence of overweight and obesity in the past decades.

The estimated demand elasticities in this study are useful information to help food policy decisionmakers understand how changes in food prices and income would affect the overall nutritional quality of American diets. Public and private sector nutritionists have increasingly advocated the need for Americans to increase their consumption of fruits and vegetables and reduce fats in their diets. However, proponents of price manipulations, such as subsidizing fruits and vegetables and taxing fats, should be aware of how economic factors influence the nutrient content of diets. Our estimated price elasticities indicate that a price reduction in fruits and vegetables could be effective in increasing produce consumption, but the effect of taxing fats to reduce fat consumption could be limited.

The estimated nutrient demand elasticities demonstrate the complexity of the effect of a change in income or price on overall diet quality. For example, a price increase for the meat group would decrease the levels of saturated fat and cholesterol, and this effect is a nutritional improvement given that these components are currently consumed in excess. However, the level of iron, which is currently consumed in insufficient amount, would decrease. Similarly, the nutritional effect of increasing consumer income is mixed. Currently insufficient intakes of nutrients, such as calcium, iron, and various vitamins, could be improved with increased incomes. Those already excessive intakes of nutrients such as energy, saturated fats, and cholesterol, however, would be exacerbated by increased incomes.

The nutrient demand elasticities could be applied for studying possible food program effects on the overall availability of nutrients. One way to accomplish this task would be to simulate alternative food policy scenarios and explore the effects of changes in food prices and income on the amount of different nutrients available for consumption. In particular, the nutrient income responses could be a starting point in evaluating possible effects of income changes on dietary quality when the benefits to food stamp recipients are cut or increased. Some adjustments, however, might be needed to reflect differences in behavior across different population groups. Also, the behavior of food spending from food stamps may be different from food spending out of money income.

## Competing interests

The views expressed here are those of the authors and cannot be attributed to the Economic Research Service or the U.S. Department of Agriculture.

## Authors' contributions

KH and SH are responsible for data compilation, result interpretation and editing. KH also contributes to model specification and estimation. Both authors read and approved the final manuscript.

## References

[B1] U.S. Surgeon General, U.S. Department of Health and Human Services The Surgeon General's Vision for a Healthy and Fit Nation 2010 http://www.surgeongeneral.gov

[B2] Dietary Guidelines Advisory Committee Report of the Dietary Guidelines Advisory Committee on the Dietary Guidelines for Americans 2010 http://www.cnpp.usda.gov/DietaryGuidelines.htm

[B3] Gawn G Innes R Rausser G Zilberman D Nutrient Demand and the Allocation of Time: Evidence from Guam Applied Economics 1993 25 811 830 10.1080/00036849300000136

[B4] Drewnowski A Darmon N The Economics of Obesity: Dietary Energy Density and Energy Cost American Journal of Clinical Nutrition 2005 82 265 273 10.1093/ajcn/82.1.265S 16002835

[B5] Allais O Bertail P Nichele V The Effects of a Fat Tax on French Households' Purchases: A Nutritional Approach American Journal of Agricultural Economics 2010 92 228 245 10.1093/ajae/aap004

[B6] Chouinard H Davis D LaFrance J Perloff J Fat Taxes: Big Money for Small Change Forum for Health Economics & Policy 2007 10 1 28

[B7] Lancaster K A New Approach to Consumer Theory Journal of Political Economy 1966 132 157

[B8] Barten A Consumer Demand Functions Under Conditions of Almost Additive Preferences Econometrica 1964 32 1 38 10.2307/1913731

[B9] Theil H The Information Approach to Demand Analysis Econometrica 1965 30 67 87

[B10] Deaton A Muellbauer J An Almost Ideal Demand System American Economic Review 1980 70 312 326

[B11] Christensen L Jorgenson D Lau L Transcendental logarithmic utility functions American Economic Review 1975 65 367 383

[B12] Huang K Nutrient Elasticities in a Complete Food Demand System American Journal of Agricultural Economics 1996 78 21 29 10.2307/1243775

[B13] Economic Research Service, U.S. Department of Agriculture Food Consumption Data System 2009 http://www.ers.usda.gov/data/foodconsumption/

[B14] Agricultural Research Service, U.S. Department of Agriculture USDA National Nutrient Database for Standard Reference, Release 16 (SR16) 2003 http://www.nal.usda.gov

[B15] Bureau of Labor Statistics, U.S. Department of Labor The Consumer Price Index (CPI), Food Items 2009 http://www.bls.gov/data/home.htm

[B16] Bureau of Economic Analysis, U.S. Department of Commerce Personal Consumption Expenditures (PCE) by Major Type of Product 2009 http://www.bea.gov/national/nipaweb/selectTable.asp?Selected=n#52/

